# Genes from the *exo–xis* region of λ and Shiga toxin-converting bacteriophages influence lysogenization and prophage induction

**DOI:** 10.1007/s00203-013-0920-8

**Published:** 2013-08-27

**Authors:** Sylwia Bloch, Bożena Nejman-Faleńczyk, Joanna M. Łoś, Sylwia Barańska, Krzysztof Łepek, Agnieszka Felczykowska, Marcin Łoś, Grzegorz Węgrzyn, Alicja Węgrzyn

**Affiliations:** 1 Department of Molecular Biology, University of Gdańsk, Wita Stwosza 59, 80-308 Gdańsk, Poland; 2 Laboratory for Molecular Vaccines, Intercollegiate Faculty of Biotechnology, University of Gdańsk and Medical University of Gdańsk, Kładki 24, 80-822 Gdańsk, Poland; 3 Institute of Physical Chemistry, Polish Academy of Sciences, Kasprzaka 44/52, 01-224 Warsaw, Poland; 4 Phage Consultants, Partyzantów 10/18, 80-254 Gdańsk, Poland; 5 Department of Microbiology, University of Szczecin, Felczaka 3c, 71-412 Szczecin, Poland

**Keywords:** Shiga toxin-converting bacteriophages, Lambdoid phages, Lysogenization, Prophage induction, *Exo–xis* region

## Abstract

The *exo–xis* region, present in genomes of lambdoid bacteriophages, contains highly conserved genes of largely unknown functions. In this report, using bacteriophage λ and Shiga toxin-converting bacteriophage ϕ24_Β_, we demonstrate that the presence of this region on a multicopy plasmid results in impaired lysogenization of *Escherichia coli* and delayed, while more effective, induction of prophages following stimulation by various agents (mitomycin C, hydrogen peroxide, UV irradiation). Spontaneous induction of λ and ϕ24_Β_ prophages was also more efficient in bacteria carrying additional copies of the corresponding *exo–xis* region on plasmids. No significant effects of an increased copy number of genes located between *exo* and *xis* on both efficiency of adsorption on the host cells and lytic development inside the host cell of these bacteriophages were found. We conclude that genes from the *exo–xis* region of lambdoid bacteriophages participate in the regulation of lysogenization and prophage maintenance.

## Introduction

The family of lambdoid bacteriophages is a group of temperate viruses infecting bacterial cells, which are characterized by a common scheme of genome organization and similar developmental regulation. There are two alternative developmental pathways of these phages, lytic—causing production of progeny virions and lysogenic—resulting in integration of the phage genome into host chromosome, thus forming lysogens, i.e., host cells bearing integrated phage genomes, called prophages. Under certain conditions causing a DNA damage in the bacterial host, a developmental switch, consisting of prophage induction, excision of its genome from the host chromosome, and entering the lytic mode of development, can occur (for reviews see Ptashne 2004; Węgrzyn and Węgrzyn 2005). The best investigated member of this family is bacteriophage λ, which has served as a model virus in molecular biology for some 60 years (Węgrzyn et al. 2012) and which is still a useful organism in studies on general biological processes (see, for example, Barańska et al. 2013).

Apart from being used in basic studies, lambdoid bacteriophages were found to play a crucial role in development of pathogenicity of specific bacterial strains. Examples are Shiga toxin-producing *Escherichia coli* (STEC), and particularly a subset of strains called enterohemorrhagic *E. coli* (EHEC). These strains may cause severe infections, leading to a relatively high level of morbidity and mortality (Gyles 2007; Hunt 2010). The significance of medical problems caused by STEC has been highlighted by the recent outbreak that occurred in Germany in 2011, which resulted in over 4,000 symptomatic infections, including over 50 fatal cases (Mellmann et al. 2011; Beutin and Martin 2012; Bloch et al. 2012; Karch et al. 2012; Werber et al. 2012).

High pathogenicity of STEC (including EHEC) depends on production of Shiga toxins (Gyles 2007; Hunt 2010; Mauro and Koudelka 2011). Genes coding for these toxins, the *stx* genes, are located on lambdoid prophages, called Stx phages (Allison 2007; Łoś et al. 2011, 2012). In all lambdoid phages, including Stx phages, the expression of vast majority of genes is strongly inhibited at the prophage state, due to the activity of the cI repressor (Ptashne 2004; Węgrzyn and Węgrzyn 2005; Riley et al. 2012). Effective transcription of the *stx* genes depends on the activity of the late phage promoter, *p*_R′_ (Wagner et al. 2001, 2002). This causes the requirement of prophage induction for production of Shiga toxins (Herold et al. 2004; Waldor and Friedman 2005). Therefore, understanding of mechanisms of regulation of this step in phage development is important for both basic knowledge and development of potential anti-STEC therapies. It is worth mentioning that although principles of the prophage induction have been described for cells lysogenized with bacteriophage λ and cultured under laboratory conditions (for reviews see Ptashne 2004; Węgrzyn and Węgrzyn 2005) and despite the fact that recent reports provided important information about regulation of lysogenization by Stx phages and induction of corresponding prophages (Aertsen et al. 2005; Bullwinkle and Koudelka 2011; Bullwinkle et al. 2012; Fogg et al. 2007, 2010, 2011, 2012; Imamovic and Muniesa 2012; Łoś et al. 2009, 2010; Murphy et al. 2008; Nejman et al. 2009, 2011; Nejman-Faleńczyk et al. 2012; Riley et al. 2012; Smith et al. 2012), our knowledge on modulation of these processes by various factors and conditions is still far from completeness.

The *b* region of lambdoid bacteriophages is dispensable for lytic development under standard laboratory conditions and contains genes of poorly understood roles. Within this region, there is a conserved genome fragment, located between *exo* and *xis* genes and transcribed from the *p*_L_ promoter, called the *exo–xis* region. It consists of several open reading frames of largely unknown functions. In bacteriophage λ, transient induction of the *p*_L_ promoter resulted in synchronization of the host cell cycle (Kourilsky and Knapp 1974). Subsequent studies indicated that expression of some genes from the *exo–xis* region caused inhibition of host DNA replication (Sergueev et al. 2002). The only report published to date on effects of genes from the *exo–xis* region on bacteriophage λ development demonstrated an impairment of lysogenization of cells overexpressing these genes due to negative regulation of cII-dependent phage promoters (Łoś et al. 2008a). Despite unknown biochemical functions of products of genes from this region, recent studies indicated that two orthologs of the λ Ea8.5 protein, encoded by a gene located between *exo* and *xis*, contain a newly discovered fused homeodomain/zinc-finger fold (Kwan et al. 2013). Interestingly, this domain, which is supposed to be able to interact with virus- and/or host-encoded regulatory proteins, could not be detected by primary sequence search methods (Kwan et al. 2013).

In summary, the *exo–xis* region contains highly conserved genes which should implicate their important functions, especially when considering viral genomes. Their roles are largely unknown, but they might potentially affect regulation of phage lysogenic development. Such regulation is crucial for expression of pathogenicity of STEC strains, as their virulence depends on Stx prophage induction. Therefore, in this work, we investigated effects of expression of genes from the *exo–xis* region of λ and one of Stx phages, ϕ24_B_, on various stages of phage development.

## Materials and methods

### Bacterial strains, bacteriophages, and plasmids

Phages ϕ24_Β_ (∆*stx2*::*cat*) (Allison et al. 2003) and λ papa (from our collection) were employed in this study. Bacteriophage suspensions were routinely stored in the TM buffer (10 mM Tris–HCl, 10 mM MgSO_4_, pH 7.2) at 4 °C. *E. coli* MG1655 strain (Jensen 1993) was the host of choice for bacteriophage infection, lysogenization, and prophage induction experiments. Plasmids are presented in Table 1.

**Table 1:** Plasmids

Plasmid	Characteristics, construction, and reference
pGAW3775tet	pBR328 derivative bearing phage λ *exo*–*xis* region (coordinates 27972–31747), tet^R^ (Łoś et al. 2008a)
pJW0tet	pGAW3775tet with phage λ *exo*–*xis* region removed, tet^R^ (Łoś et al. 2008a)
pJWea8.5	pJW0tet bearing the *ea8.5* gene from phage λ *exo–xis* region, tet^R^ (Łoś et al. 2008a)
pJWea22	pJW0tet bearing the *ea22* gene from phage λ *exo–xis* region, tet^R^ (Łoś et al. 2008a)
pJWorf	pJW0tet bearing *orf61*, *orf60a* and *orf63* open reading frames from phage λ *exo–xis* region, tet^R^ (Łoś et al. 2008a)
pJWorfea22	pJW0tet bearing *orf61*, *orf60a* and *orf63* open reading frames and *ea22* gene from phage λ *exo–xis* region, tet^R^ (Łoś et al. 2008a)
pJWea22ea8.5	pJW0tet bearing *ea22* and *ea8.5* genes from phage λ *exo–xis* region, tet^R^ (Łoś et al. 2008a)
pSBe.x.r.ϕ24_B_	As pGAW3775tet but bearing the *exo–xis* region from phage ϕ24_B_ instead of the homologous λ DNA fragment (constructed by replacing the StuI–BamHI fragment of pGAW3775tet with a corresponding fragment of the phage ϕ24_B_ genome encompassing the *exo–xis* region (coordinates 52853–56119), tet^R^ (this study)

For construction of pSBe.x.r.ϕ24_B_, the *exo–xis* region from phage ϕ24_B_ was amplified by PCR, using primers Φ24BStuI (5′-TGA AGG CCT GCA TTA TGT CGT GAT TGA G) and Φ24BBamHI (5′-CGG GGA TCC AGT TGA TTT CCA TAG TAT GC), and the phage genome as a template (phage ϕ24_B_ DNA was isolated using MasterPure™ Complete DNA and RNA Purification Kit; Epicentre). Following digestion with BamHI and StuI, the ϕ24_B_ *exo–xis* region was ligated with the BamHI–StuI fragment of plasmid pGAW3775tet (Łoś et al. 2008a) bearing a tetracycline resistance gene. In a series of pGAW3775tet-derived plasmids, *exo* and *xis* genes are truncated, thus non-functional (Łoś et al. 2008a). The pSBe.x.r.ϕ24_B_ plasmid contains last 126 base pairs (from the 3′ end) of the *exo* gene and only first 12 base pairs (from the 5′ end) of the *xis* gene. Therefore, no active products of *exo* and *xis* can appear due to expression of these truncated genes.

### Prophage induction experiments

Bacteria lysogenic for tested phages were cultured in Luria–Bertani (LB) medium at 37 °C to *A*_600_ of 0.1. Three induction agents were tested: 0.2 μg/ml mitomycin C, 50 J/m^2^ UV irradiation, and 1 mM H_2_O_2_. At indicated times after induction (every 30 min), samples of bacterial cultures were harvested, and 30 μl of chloroform were added to 0.5 ml of each sample. The mixture was vortexed and centrifuged for 5 min in a microcentrifuge. Then, serial dilutions were prepared in TM buffer (10 mM Tris–HCl, 10 mM MgSO_4_; pH 7.2). Phage titer (number of phages per ml) was determined by spotting 2.5 μl of each dilution of the phage lysate on a freshly prepared LB agar (1.5 %) or LB agar (1.5 %) with 2.5 μg/ml chloramphenicol (according to a procedure described by Łoś et al. 2008b), with a poured mixture of 1-ml indicator *E. coli* MG1655 strain culture and 2 ml of 0.7 % nutrient agar (prewarmed to 45 °C), supplemented with MgSO_4_ and CaCl_2_ (to a final concentration of 10 mM each). Plates were incubated at 37 °C overnight. Analogous experiments but without induction agents were performed (control experiments) with each lysogenic strain. Presented values show phage titer (PFU/ml) normalized to results of control experiments (representing ratios of phage titers in induced and non-induced cultures). Each experiment was repeated three times.

### One-step-growth experiment

Lytic development of lambdoid phages was studied in one-step-growth experiments. Bacteria were grown in LB medium supplemented with MgSO_4_ and CaCl_2_ (to a final concentration of 10 mM each) at 37 °C to *A*_600_ = 0.2. Samples of 10 ml were withdrawn and centrifuged (3,000×*g*, 10 min). Each pellet was suspended in 1 ml (1/10 of initial volume) of 3 mM NaN_3_ in LB. Following 5-min incubation at 37 °C, the phage was added to multiplicity of infection (m.o.i.) of 0.05. Phage adsorption was carried out at 37 °C for 10 min. The mixture was diluted tenfold in warm (37 °C) 3 mM NaN_3_ in LB and centrifuged (3,000×*g*, 10 min). Bacterial pellet was suspended in 1 ml of LB with 3 mM NaN_3_ and centrifuged again (3,000×*g*, 10 min). This procedure was repeated three times. The suspension was then diluted 1,000-fold with LB, prewarmed to 37 °C (time 0), and aerated in a water bath shaker at this temperature. The number of infective centers was estimated from nine samples taken in the interval of 0–15 min after the dilution by plating under permissive conditions. The number of intracellular progeny phages (samples previously shaken vigorously for 1 min with equal volume of chloroform and cleared by centrifugation) was estimated by plating on appropriate indicator bacteria. Plates were incubated at 37 °C overnight. Each experiment was repeated three times.

### Efficiency of lysogenization

Host bacteria were cultured to *A*_600_ = 0.5 in LB medium supplemented with MgSO_4_ and CaCl_2_ (to a final concentration of 10 mM each) at 37 °C with shaking. Aliquots of these cultures were mixed with phage suspensions at m.o.i. of 1, 5 or 10 in a final volume of 100 μl. After 30-min incubation at 37 °C, serial dilutions were prepared in TM buffer (10 mM Tris–HCl, 10 mM MgSO_4_; pH 7.2) and the mixture was plated onto LB agar (control) and selective medium LB containing 20 μg/ml chloramphenicol (presumptive lysogens). Plates were incubated at 37 °C overnight. Lysogens were verified by testing resistance to superinfection by the same phage and sensitivity to UV irradiation, as described previously (Wegrzyn et al. 1992). Each experiment was repeated three times.

### Measurement of the efficiency of phage adsorption

Bacteria were grown in LB medium supplemented with MgSO_4_ and CaCl_2_ (to a final concentration of 10 mM each) at 37 °C to *A*_600_ = 0.2–0.4, and bacteriophages were added to m.o.i. of 0.1. The mixture was incubated at 37 °C. At indicated times, samples were withdrawn, centrifuged 1 min in a microcentrifuge, and the supernatant was titrated. Plates were incubated at 37 °C overnight. Each experiment was repeated three times. A sample withdrawn immediately after addition of bacteriophages to the cell suspension (time zero) was considered as 100 % non-adsorbed phages. Other values were calculated relative to this value.

### Survival of cells after bacteriophage infection

Host bacteria were grown in LB medium at 37 °C to *A*_600_ = 0.3. Samples of 4 ml were centrifuged and pellets were washed with 1 ml of 0.9 % NaCl. After centrifugation, each pellet was suspended in 1.2 ml of LB medium supplemented with MgSO_4_ and CaCl_2_, to a final concentration of 10 mM each. The mixture was incubated for 30 min at 37 °C. Bacteriophage lysate was added to m.o.i. of 1, 5 or 10. Following 30 min incubation at 37 °C, serial dilutions in TM buffer were prepared. 30 μl of each dilution was platted on LB agar plates and incubated at 37 °C overnight. Fraction of surviving bacteria was calculated relative to the parallel sample with addition of TM buffer instead of bacteriophage lysate. Each experiment was repeated three times.

## Results

### Comparison between the *exo–xis* regions from λ and Stx bacteriophages

**Fig. 1:**
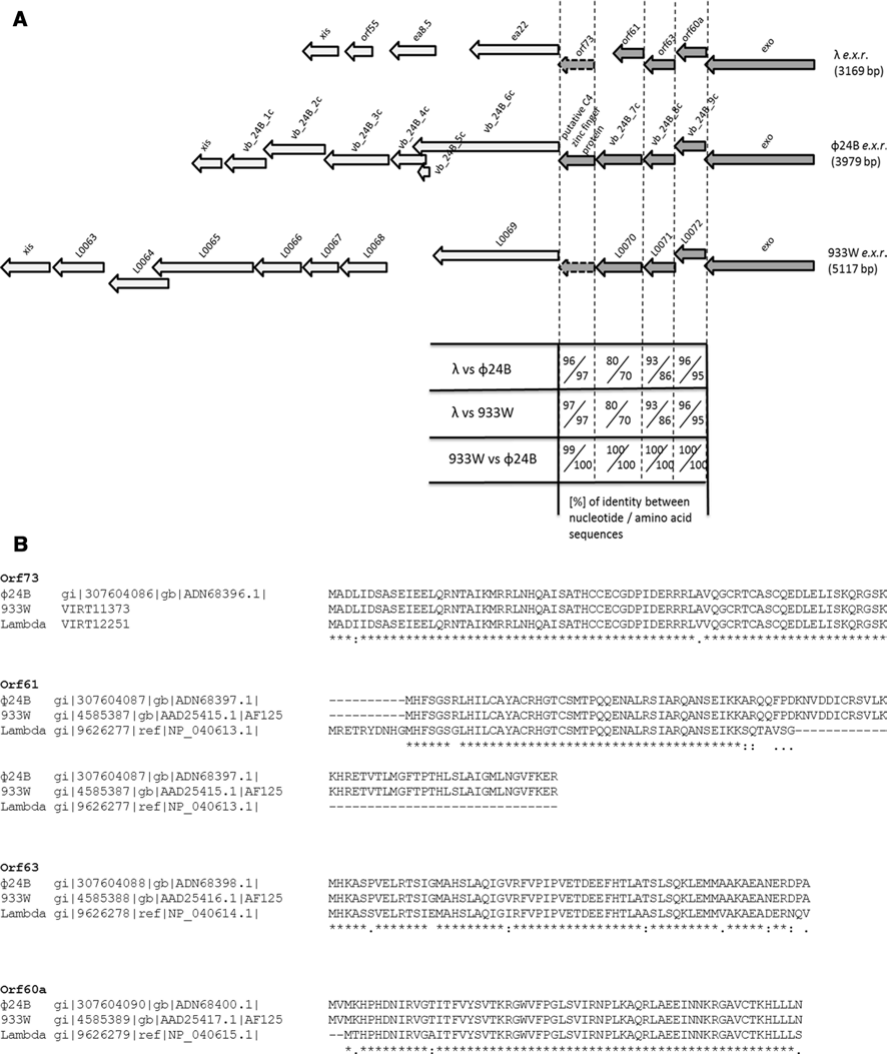
Comparison between sequences of open reading frames (**a**) and their putative products (**b**) from the *exo–xis* regions (*e.x.r*.) of bacteriophages λ, ϕ24_B_ and 933W (accession numbers: GI:9626243, GI:307604077, GI:4585377, respectively). **a** *Dark arrows* with *continuous outer border lines* represent highly conserved (>70 % sequence identity) genes and open reading frames. *Dark arrows* with *punctuated outer borders* represent highly conserved (>70 % sequence identity) open reading frames present in genomes of λ and 933W phages, which are available in the NCBI database but were either uncharacterized or even not mentioned in annotations. The presence of *orf73* in the λ *exo–xis* region was indicated by Sergueev et al. (2002). *Light arrows* represent genes and open reading frames with low level (<35 %) of identity. The pairwise scores were calculated for every pair of sequences that was aligned using the ClustalW program, but only highly conserved (>70 % sequence identity) homologs of λ *orf60a*, *orf 63*, *orf61*, and *orf73* were considered. Pairwise scores represent the number of identities between two compared sequences, divided by the length of the alignment, and shown as a percentage. **b** ClustalW program was used to align multiple amino acid sequences. Translation of nucleotide sequences of λ *orf73* and its 933W homolog were generated on ExPASy, and VIRT12251 and VIRT11373 products were predicted, respectively

The *exo–xis* region of bacteriophage λ genome, previously described by Sergueev et al. (2002), contains 7 open reading frames, named: *orf60a*, *orf63*, *orf61*, *orf73*, *ea22*, *ea8.5* and *orf55*. Comparatively, *exo–xis* regions of genomes of a couple of the best investigated Stx phages, ϕ24_B_ and 933W, contain additional open reading frames (Fig. 1). We found that four of the identified ORFs are highly conserved among λ and tested Stx phages. Genomes of phages ϕ24_B_ and 933W contain homologs of following phage λ ORFs: *orf60a*, *orf63*, *orf61*, and *orf73* which reveal high levels (>70 %) of identity of DNA sequences and amino acid sequences of putative gene products (Fig. 1). Other ORFs shown in Fig. 1 were of low similarity (<35 % of sequence identity at DNA and protein levels).

### The ϕ24_B_*exo–xis* region impairs lysogenization when present on a plasmid

It was demonstrated previously that the presence of the λ *exo–xis* region on a multicopy plasmid resulted in a decreased efficiency of lysogenization by this bacteriophage (Łoś et al. 2008a). To test effects of the ϕ24_B_ *exo–xis* region on development of this Stx phage, a ColE1-like plasmid bearing appropriate DNA fragment, pSBe.x.r.ϕ24_B_, has been constructed (Table 1). In control experiments, a bacterial strain bearing the pJW0tet vector (a plasmid analogous to pSBe.x.r.ϕ24_B_ but devoid of the *exo–xis* region) was used.

We found that *E. coli* cells bearing a multicopy plasmid with the ϕ24_B_ *exo–xis* region (pSBe.x.r.ϕ24_B_) were lysogenized less efficiently by the ϕ24_B_ bacteriophage than bacteria bearing the vector (Table 2). This effect was similar to that observed previously for bacteriophage λ (Łoś et al. 2008a).

**Table 2:** Effects of the ϕ24_B_ *exo–xis* region on lysogenization of *E. coli* cells by phage ϕ24_B_

Plasmid in *E. coli* host	Efficiency of lysogenization (% of lysogens among survivors)^a^		
	m.o.i. = 1	m.o.i. = 5	m.o.i. = 10
pJW0tet (vector)	22 ± 0.4	87 ± 6.4	65 ± 5.0
pSBe.x.r.ϕ24_B_ (*exo–xis* region of ϕ24_B_)	12 ± 0.9	21 ± 1.0	13 ± 0.4

^a^Mean values from three independent experiments ±SD are shown

### Lytic development of the ϕ24_B_ is not affected by the presence of the *exo–xis* region on a multicopy plasmid

**Fig. 2:**
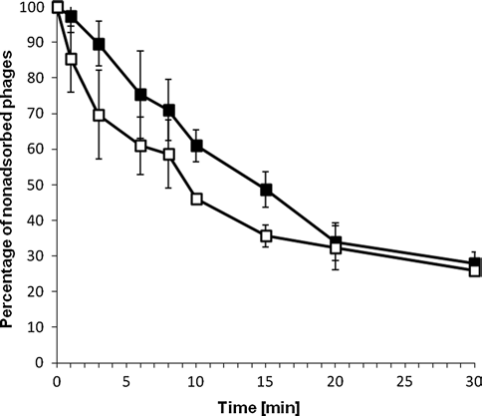
Adsorption of bacteriophage ϕ24_B_ on *E. coli* MG1655 host bearing the pJW0tet vector (*closed squares*) or the pSBe.x.r.ϕ24_B_ plasmid (*open squares*) which contains the *exo–xis* region of ϕ24_B_. The presented results are mean values from three experiments with SD indicated by *error bars*

**Fig. 3:**
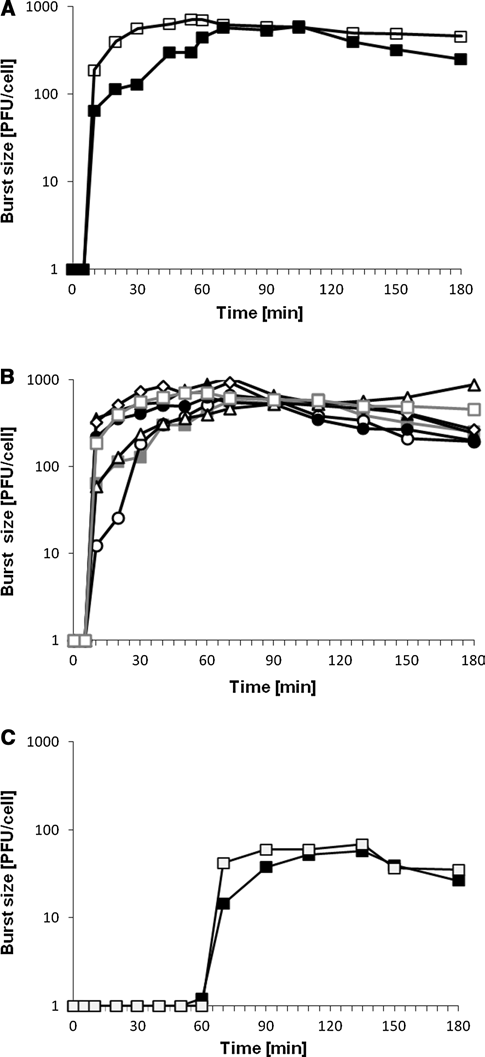
Lytic development, assessed in one-step-growth experiments, of bacteriophages λ (**a**, **b**) and ϕ24_B_ (**c**) in the *E. coli* MG1655 host bearing different plasmids. *Symbols* in diagrams denote host cells which bear following plasmids: **a** pJW0tet (*closed squares*), pGAW3775tet (*open squares*); **b** pJW0tet (*closed squares*), pGAW3775tet (*open squares*), pJWea8.5 (*open diamonds*), pJWea22 (*closed triangles*), pJWorf (*open circles*), pJWorfea22 (*closed circles*), pJWea22ea8.5 (*open triangles*); **c** pJW0tet (*closed squares*), pSBe.x.r.ϕ24_B_ (*open squares*). Results are shown as PFU (plaque forming units) per cell. The presented results are mean values from three experiments. SD was below 20 % for each point, and is not shown for clarity of presentation

To test potential effects of the *exo–xis* region on lytic development of phage ϕ24_B_, we have measured efficiency of adsorption of the phage on host cells and kinetics of phage progeny formation in the presence and absence of the *exo–xis* region on a multicopy plasmid. We found that neither adsorption of ϕ24_B_ on *E. coli* cells nor its intracellular lytic development (assessed by measurement of burst size) was affected by the presence of the pSBe.x.r.ϕ24_B_ plasmid (Figs. 2, 3c, respectively). These results were analogous to those reported previously for bacteriophage λ (Łoś et al. 2008a). Interestingly, while the *exo–xis* region of bacteriophage λ was reported to enhance survival of *E. coli* cells in cultures infected with λ (Łoś et al. 2008a), an opposite effect was observed when bacteria bearing the pSBe.x.r.ϕ24_B_ plasmid were infected with phage ϕ24_B_, i.e., lower number of cells survived relative to the strain bearing the plasmid vector (Table 3).

**Table 3:** Effects of the ϕ24_B_ *exo–xis* region on survival of *E. coli* cells after infection with phage ϕ24_B_

Plasmid in *E. coli* host	Survival of cells in infected culture (% of survivors)^a^		
	m.o.i. = 1	m.o.i. = 5	m.o.i. = 10
pJW0tet (vector)	100 ± 5.3	28 ± 3.0	30 ± 3.0
pSBe.x.r.ϕ24_B_ (*exo–xis* region of ϕ24_B_)	36 ± 7.9	10 ± 3.0	6 ± 1.1

^a^Mean values from three independent experiments ±SD are shown

### Effects of the *exo–xis* region on induction of λ and ϕ24_B_ prophages

To test effects of the *exo–xis* region on prophage induction, we have employed lysogenic cells bearing either a multicopy plasmid with this region or a plasmid vector. Since efficiency of prophage induction in hosts containing additional copies of the *exo–xis* region were not tested previously for λ, we have used this phage along with phage ϕ24_B_, and employed plasmids carrying corresponding *exo–xis* regions (pGAW3775tet and pSBe.x.r.ϕ24_B_, respectively). We have applied different known inducers of lambdoid prophages (mitomycin C, hydrogen peroxide, and UV irradiation) and estimated number of progeny phages appearing at various times after induction. Knowing that *exo–xis* regions of λ and ϕ24_B_ do not influence burst size of both tested phages (Fig. 3), we assumed that kinetics of appearance of progeny viruses should reflect efficiency of prophage induction.

**Fig. 4:**
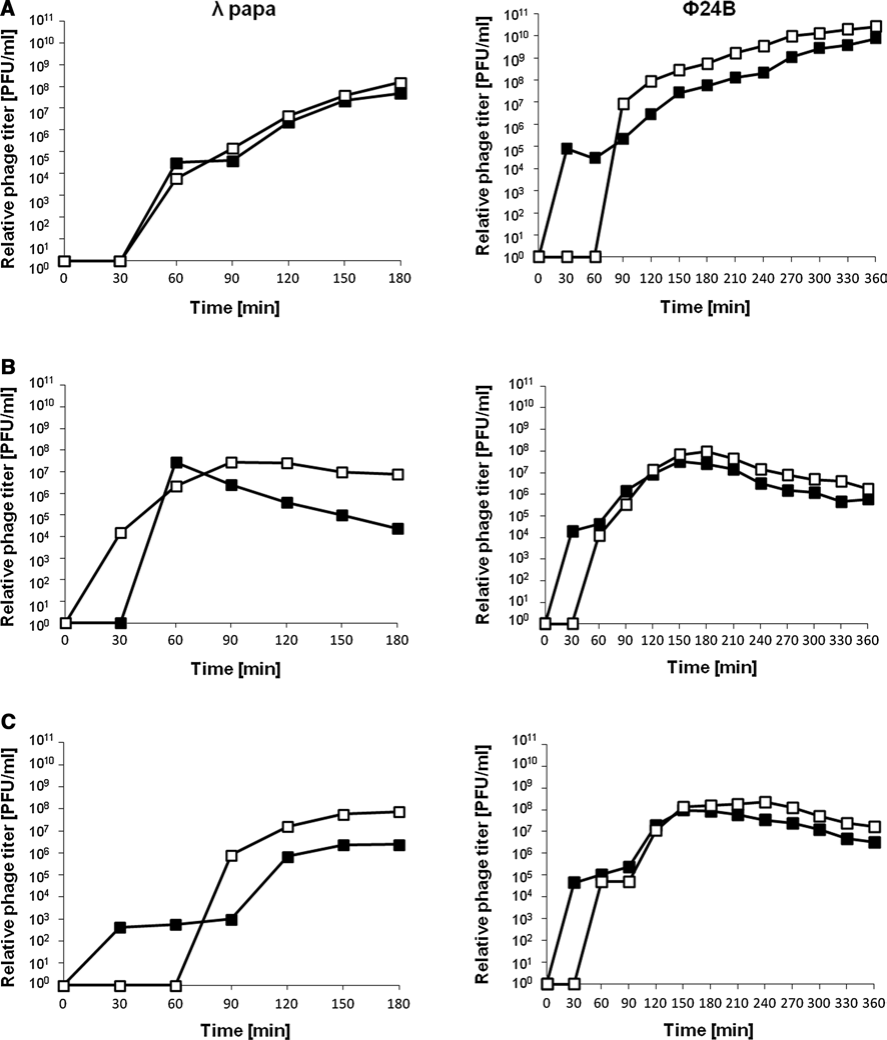
Induction of prophages λ (*left-side diagrams*) and ϕ24_B_ (*right-side diagrams*) in MG1655 hosts bearing the pJW0tet vector (*closed squares*) or either pGAW3775tet (*left-side diagrams*) or pSBe.x.r.ϕ24_B_ (*right-side diagrams*) (*open squares*), treated with 0.2 μg/ml mitomycin C (**a**), 50 J/m^2^ UV irradiation (**b**), or 1 mM H_2_O_2_ (**c**) at time 0. Results are shown as PFU (plaque forming units) per cell. The presented results are mean values from three experiments. SD was below 20 % for each point, and is not shown for clarity of presentation

**Fig. 5:**
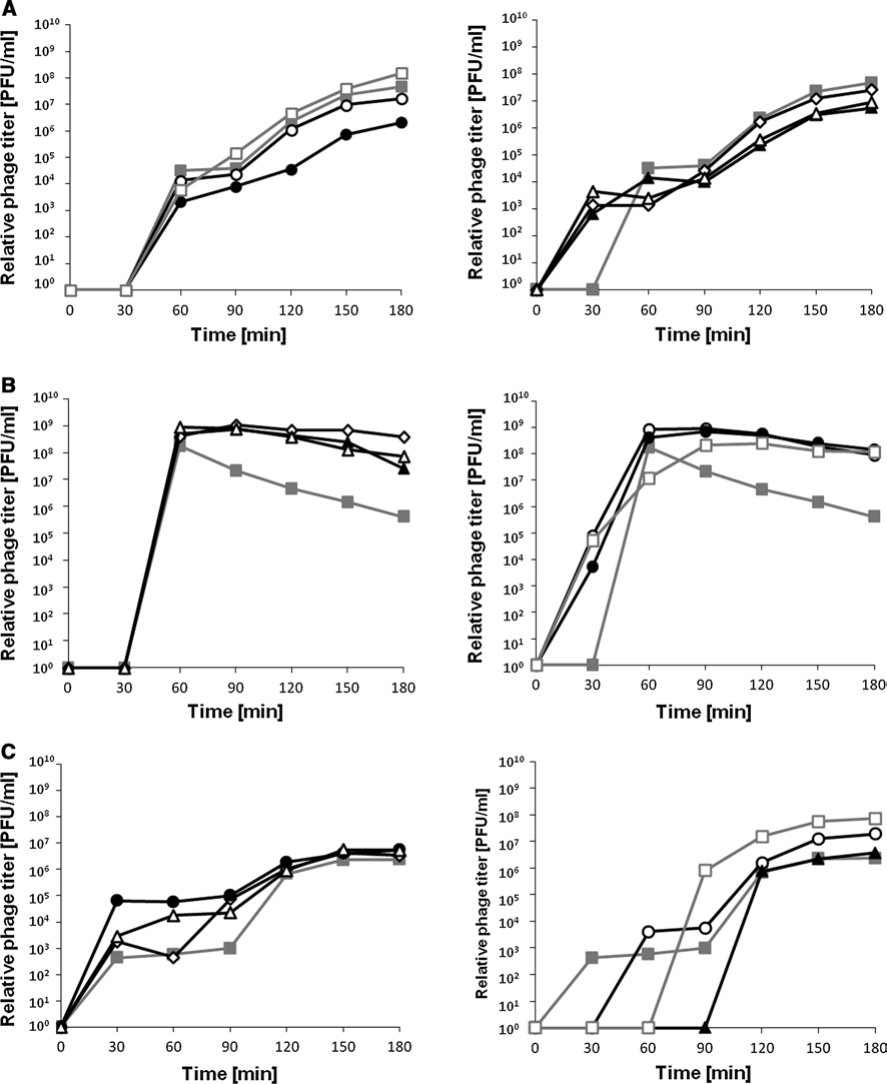
Induction of prophage λ in MG1655 hosts bearing the pJW0tet vector (*closed squares*), pGAW3775tet (*open squares*), pJWea8.5 (*open diamonds*), pJWea22 (*closed triangles*), pJWorf (*open circles*), pJWorfea22 (*closed circles*) or pJWea22ea8.5 (*open triangles*). Bacterial cultures were treated with 0.2 μg/ml mitomycin C (**a**), 50 J/m^2^ UV irradiation (**b**), or 1 mM H_2_O_2_ (**c**) at time 0. Results are shown as PFU (plaque forming units) per cell. The presented results are mean values from three experiments. SD was below 20 % for each point, and is not shown for clarity of presentation

We found that induction of the ϕ24_B_ prophage was delayed by 30–60 min in the presence of the *exo–xis* region on a multicopy plasmid, but this process was finally more efficient than that in control experiments, as more progeny phages were produced (Fig. 4). This phenomenon occurred irrespective of the kind of the inducer used, nevertheless, the delay in prophage induction was the longest (60 min) in experiments with mitomycin C (Fig. 4). Contrary to ϕ24_B_, effects of the *exo–xis* region on λ prophage induction depended on the nature of the inducing agent. The presence of the pGAW3775tet plasmid had no effect relative to the vector when mitomycin C was used, delayed the induction in the presence of hydrogen peroxide, and caused earlier induction after UV irradiation (Fig. 4). Nevertheless, with exception of the induction with mitomycin C, final efficiency of induction of λ prophage was higher in the presence of the *exo–xis* region on a plasmid relative to control experiments (Fig. 4). When particular genes or combinations of two or a few genes from the λ *exo–xis* region were present on the plasmid, their effect on λ prophage induction was generally less pronounced than that of the whole region (Fig. 5).

We have also estimated efficiency of spontaneous (without any induction agent) induction of prophages λ and ϕ24_B_ to find that this parameter is significantly elevated in the presence of corresponding *exo–xis* regions on multicopy plasmids relative to plasmid vectors (Table 4). Therefore, extra copies of the *exo–xis* regions destabilized λ and ϕ24_B_ prophages.

**Table 4:** Efficiency of spontaneous induction of λ and ϕ24_B_ prophages in the presence of corresponding *exo–xis* regions on multicopy plasmids

Plasmid in *E. coli* host	Efficiency of spontaneous prophage induction (induction events per cell)^a^	
	λ	ϕ24_B_
pJW0tet (vector)	3.2 × 10^−7^ ± 1.0 × 10^−8^	4.0 × 10^−5^ ± 3.1 × 10^−7^
pGAW3775tet (*exo–xis* region of λ)	3.2 × 10^−6^ ± 7.5 × 10^−8^	nd
pSBe.x.r.ϕ24_B_ (*exo–xis* region of ϕ24_B_)	nd	4.4 × 10^−4^ ± 8.0 × 10^−6^

^a^Mean values from three independent experiments ±SD are shown. The values were calculated considering number of infective virions appearing in the growth medium of cultures of lysogenic bacteria and average burst size of the phage estimated in one-step-growth experiments

*nd* Not determined

## Discussion

Pathogenicity of enterohemorrhagic *E. coli* strains strongly depends on production and release of Shiga toxins (Mauro and Koudelka 2011). Expression of genes coding for these toxins requires induction of lambdoid prophages (Gyles 2007; Hunt 2010). Moreover, it appears that a biological role for production of Shiga toxins by *E. coli* may be ascribed to killing unicellular eukaryotic predators, while toxicity to humans was speculated to be a side effect of an attack by human neutrophils which produce hydrogen peroxide, thus potentially causing Stx prophage induction (Łoś et al. 2011, 2012; Mauro and Koudelka 2011). It was demonstrated recently that Shiga toxin released inside the eukaryotic cell as a consequence of digestion of bacteria by a protist is harmless to the predator (Stolfa and Koudelka 2012). This implies that bacteriophage-mediated lysis of the host bacterium is necessary for the toxicity to the predator. Knowing the mechanism of Shiga toxin action (for a review see Mauro and Koudelka 2011, and references therein), one may suggest that binding of the toxin to its receptor on the cell membrane ensures not only its further effective transport inside the cell, but also its modification and retrotranslocation to the cytoplasm, where it can inactivate ribosomes; this might be impossible if the toxin is released inside the cell. Obviously, to achieve a benefit from production of Shiga toxins, *E. coli* cells lysogenic for Stx phages must be ensured that frequency of prophage induction is limited sufficiently enough to allow survival of a large fraction of the population while scarifying some cells, acting as “altruists” in order to kill the predator and to save the rest of bacteria (Łoś et al. 2012).

In the light of the biology and pathogenicity of Shiga toxin-producing *E. coli*, summarized above, it is clear that regulation of Stx prophage maintenance and induction is a crucial process for these organisms. On the other hand, while induction of λ prophage under laboratory conditions is relatively well understood, the control of such a process occurring in lambdoid prophages which persist in natural environments remains unclear. In this light, it was intriguing to investigate roles of genes located in the *exo–xis* regions of genomes of lambdoid prophages. Among these genes, four are highly conserved, but their functions for bacteriophage development remain largely unknown. In this report, we provide evidence that the presence of the ϕ24_B_ *exo–xis* region on a multicopy plasmid in the host cell results in a decreased efficiency of lysogenization by this phage (similarly to what has already been demonstrated for bacteriophage λ by Łoś et al. 2008a) and in delayed, while more efficient, induction of the corresponding prophage by various agents (mitomycin C, hydrogen peroxide, UV irradiation). Moreover, the presence of additional copies of the *exo–xis* region caused destabilization of the ϕ24_B_ prophage in the absence of any external inducing agents. Effects of the *exo–xis* region on prophage induction and stability were also evident for λ. It is worth noting that the experimental system used in this work compared effects of the presence of additional copies of the *exo–xis* region, located on multicopy plasmids, versus single copies of the investigated genes located in genomes of bacteriophages, either infecting host cells or being integrated into host chromosomes in lysogens. On the one hand, this is an advantage since it allows to observe the effects of gene dosage, but on the other hand, this may cause less pronounced differences in experiments where various m.o.i. conditions are used, like in assessment of lysogenization efficiency. In the latter case, while impairment of lysogenization of cells bearing multicopy plasmids with the *exo–xis* region is evident relative to the control system, the effects of various m.o.i. values are relatively minor in the presence of additional *exo–xis* copies on plasmids.

In most cases, effects of additional copies of the *exo–xis* region are similar between λ and Stx phages. The biggest difference occurs in the influence of plasmids bearing this region on bacterial survival following bacteriophage infection. While *E. coli* survives the infection by λ more efficiently at increased doses of genes from the *exo–xis* region, the presence of additional copies of a homologous region from the ϕ24_B_ phage resulted in a decreased bacterial survival in infected cultures. Interestingly, kinetics of production of progeny phages was not significantly affected by the *exo–xis* region in both λ and ϕ24_B_. In this light, it is worth reminding that bacteriophages can kill their hosts irrespective of completing their lytic growth and causing cell lysis (for reviews see Ptashne 2004; Węgrzyn et al. 2012). It is likely that the observed effects on bacterial survival are related to previously reported modulation of regulation of host cell cycle and DNA replication by activities of some genes located between *exo* and *xis* (Kourilsky and Knapp 1974; Sergueev et al. 2002). If so, it is tempting to speculate that the genes of low similarity rather than the highly conserved genes from the *exo–xis* regions are responsible for the differences between λ and ϕ24_B_.

The results presented in this report clearly demonstrate a role for the *exo–xis* region in the regulation of lambdoid prophage maintenance. The molecular mechanisms of actions of particular genes or their products in this phenomenon remain unknown. However, results of experiments with either separate genes or gene clusters from the λ *exo–xis* region suggest that their products cooperate in order to fully express their control function in prophage induction. The only gene from this region for which biochemical activity of its product can be predicted is *ea8.5*. Recent structural studies indicated that the Ea8.5 protein can potentially interact with some regulatory proteins (Kwan et al. 2013). Therefore, one may speculate that putative interactions between this protein and cI or cII transcription regulators, which are major players in the control of prophage maintenance and lysogenization, respectively, could significantly influence these processes in lambdoid phages. In fact, modulation of cII-dependent transcription stimulation by an increased gene dosage of either *ea8.5* or the whole *exo–xis* region has already been demonstrated experimentally (Łoś et al. 2008a). Undoubtedly, characterization of properties of other proteins encoded in this region will be necessary to understand molecular mechanisms of phenomena described in this report and in previous articles which indicated considerable effects of genes located between *exo* and *xis* on both lambdoid phage development and physiology of the host cell (Kourilsky and Knapp 1974; Sergueev et al. 2002; Łoś et al. 2008a).

What are mechanisms and biological significance for the *exo–xis* region-mediated modulation of the efficiency of Stx prophage induction demonstrated for the first time in this work? During lysogeny, the phage-encoded cI protein represses *p*_L_ promoter-directed transcription of the *exo–xis* region. Therefore, it is tempting to speculate that any leakiness of this repression may result in expression of genes from this region and promotion of prophage induction. The efficiency of such a process would perhaps be low; however, it might be sufficient to achieve the level observed during the attack of either protozoan predators or human neutrophils, when induction of Stx prophages in a few percent of cells may ensure production of large amounts of toxins. This, in turn, might result in either survival of the bacterial population or expression of STEC pathogenicity, respectively.

## References

[CR1] Aertsen A, Faster D, Michiels CW (2005). Induction of Shiga toxin-converting prophage in *Escherichia coli* by high hydrostatic pressure. Appl Environ Microbiol.

[CR2] Allison HE (2007). Stx-phages: drivers and mediators of the evolution of STEC and STEC-like pathogens. Future Microbiol.

[CR3] Allison HE, Sergeant MJ, James CE, Saunders JR, Smith DL, Sharp RJ, Marks TS, McCarthy AJ (2003). Immunity profiles of wild-type and recombinant Shiga-like toxin-encoding bacteriophages and characterization of novel double lysogens. Infect Immun.

[CR4] Barańska S, Glinkowska M, Herman-Antosiewicz A, Maciąg-Dorszyńska M, Nowicki D, Szalewska-Pałasz A, Węgrzyn A, Węgrzyn G (2013). Replicating DNA by cell factories: roles of central carbon metabolism and transcription in the control of DNA replication in microbes, and implications for understanding this process in human cells. Microb Cell Fact.

[CR5] Beutin L, Martin A (2012). Outbreak of Shiga toxin-producing *Escherichia coli* (STEC) O104:H4 infection in Germany causes a paradigm shift with regard to human pathogenicity of STEC strains. J Food Prot.

[CR6] Bloch S, Felczykowska A, Nejman-Faleńczyk B (2012). *Escherichia coli* O104:H4 outbreak—have we learnt a lesson from it?. Acta Biochim Pol.

[CR7] Bullwinkle TJ, Koudelka GB (2011). The lysis-lysogeny decision of bacteriophage 933W: a 933W repressor-mediated long-distance loop has no role in regulating 933W P(RM) activity. J Bacteriol.

[CR8] Bullwinkle TJ, Samorodnitsky D, Rosati RC, Koudelka GB (2012). Determinants of bacteriophage 933W repressor DNA binding specificity. PLoS ONE.

[CR9] Fogg PCM, Gossage SM, Smith DL, Saunders JR, McCarthy AL, Allison HE (2007). Identification of multiple integration sites for Stx-phage φ24_B_ in the *Escherichia coli* genome, description of a novel integrase and evidence for a functional anti-repressor. Microbiology.

[CR10] Fogg PC, Allison HE, Saunders JR, McCarthy AJ (2010). Bacteriophage lambda: a paradigm revisited. J Virol.

[CR11] Fogg PC, Rigden DJ, Saunders JR, McCarthy AJ, Allison HE (2011). Characterization of the relationship between integrase, excisionase and antirepressor activities associated with a superinfecting Shiga toxin encoding bacteriophage. Nucleic Acids Res.

[CR12] Fogg PC, Saunders JR, McCarthy AJ, Allison HE (2012). Cumulative effect of prophage burden on Shiga toxin production in *Escherichia coli*. Microbiology.

[CR13] Gyles CL (2007). Shiga toxin-producing *Escherichia coli*: an overview. J Anim Sci.

[CR14] Herold S, Karch H, Schmidt H (2004). Shiga toxin-encoding bacteriophages—genomes in motion. Int J Med Microbiol.

[CR15] Hunt JM (2010). Shiga toxin-producing *Escherichia coli* (STEC). Clin Lab Med.

[CR16] Imamovic L, Muniesa M (2012). Characterizing RecA-independent induction of Shiga toxin2-encoding phages by EDTA treatment. PLoS ONE.

[CR17] Jensen KF (1993). The *Escherichia coli* K-12 “wild types” W3110 and MG1655 have an *rph* frameshift mutation that leads to pyrimidine starvation due to low *pyrE* expression levels. J Bacteriol.

[CR18] Karch H, Denamur E, Dobrindt U, Finlay BB, Hengge R, Johannes L, Ron EZ, Tønjum T, Sansonetti PJ, Vicente M (2012). The enemy within us: lessons from the 2011 European *Escherichia coli* O104:H4 outbreak. EMBO Mol Med.

[CR19] Kourilsky P, Knapp A (1974). Lysogenization by bacteriophage λ. III. Multiplicity dependent phenomena occurring upon infection by λ. Biochimie.

[CR20] Kwan JJ, Smirnova E, Khazai S, Evanics F, Maxwell KL, Donaldson LW (2013). The solution structures of two prophage homologues of the bacteriophage λ Ea8.5 protein reveal a newly discovered hybrid homeodomain/zinc-finger fold. Biochemistry.

[CR21] Łoś JM, Łoś M, Wegrzyn A, Wegrzyn G (2008). Role of the bacteriophage λ *exo–xis* region in the virus development. Folia Microbiol.

[CR22] Łoś JM, Golec P, Węgrzyn G, Węgrzyn A, Łoś M (2008). Simple method for plating *Escherichia coli* bacteriophages forming very small plaques or no plaques under standard conditions. Appl Environ Microbiol.

[CR23] Łoś JM, Łoś M, Węgrzyn G, Węgrzyn A (2009). Differential efficiency of induction of various lambdoid prophages responsible for production of Shiga toxin in response to different induction agents. Microb Pathog.

[CR24] Łoś JM, Łoś M, Węgrzyn A, Węgrzyn G (2010). Hydrogen peroxide-mediated induction of the Shiga toxin-converting lambdoid prophages ST2-8624 in *Escherichia coli* O157:H7. FEMS Immunol Med Microbiol.

[CR25] Łoś JM, Łoś M, Węgrzyn G (2011). Bacteriophages carrying Shiga toxin genes: genomic variations, detection and potential treatment of pathogenic bacteria. Future Microbiol.

[CR26] Łoś JM, Łoś M, Węgrzyn A, Węgrzyn G (2012). Altruism of Shiga toxin-producing *Escherichia coli*: recent hypothesis versus experimental results. Front Cell Infect Microbiol.

[CR27] Mauro SA, Koudelka GB (2011). Shiga toxin: expression, distribution, and its role in the environment. Toxins.

[CR28] Mellmann A, Harmsen D, Cummings CA, Zentz EB, Leopold SR, Rico A, Prior K, Szczepanowski R, Ji Y, Zhang W, McLaughlin SF, Henkhaus JK, Leopold B, Bielaszewska M, Prager R, Brzoska PM, Moore RL, Guenther S, Rothberg JM, Karch H (2011). Prospective genomic characterization of the German enterohemorrhagic *Escherichia coli* O104:H4 outbreak by rapid next generation sequencing technology. PLoS ONE.

[CR29] Murphy KC, Ritchie JM, Waldor MK, Løbner-Olesen A, Marinus MG (2008). Dam methyltransferase is required for stable lysogeny of the Shiga toxin (Stx2)-encoding bacteriophage 933W of enterohemorrhagic *Escherichia coli* O157:H7. J Bacteriol.

[CR30] Nejman B, Łoś JM, Łoś M, Węgrzyn G, Węgrzyn A (2009). Plasmids derived from lambdoid bacteriophages as models for studying replication of mobile genetic elements responsible for the production of Shiga toxins by pathogenic *Escherichia coli* strains. J Mol Microbiol Biotechnol.

[CR31] Nejman B, Nadratowska-Wesołowska B, Szalewska-Pałasz A, Węgrzyn A, Węgrzyn G (2011). Replication of plasmids derived from Shiga toxin-converting bacteriophages in starved *Escherichia coli*. Microbiology.

[CR32] Nejman-Faleńczyk B, Golec P, Maciąg M, Węgrzyn A, Węgrzyn G (2012). Inhibition of development of Shiga toxin-converting bacteriophages by either treatment with citrate or amino acid starvation. Foodborne Pathog Dis.

[CR33] Ptashne M (2004). A genetic switch: phage lambda revisited.

[CR34] Riley LM, Veses-Garcia M, Hillman JD, Handfield M, McCarthy AJ, Allison HE (2012). Identification of genes expressed in cultures of *E. coli* lysogens carrying the Shiga toxin-encoding prophage Φ24B. BMC Microbiol.

[CR35] Sergueev K, Court D, Reaves L, Austin S (2002). *E. coli* cell-cycle regulation by bacteriophage λ. J Mol Biol.

[CR36] Smith DL, Rooks DJ, Fogg PC, Darby AC, Thomson NR, McCarthy AJ, Allison HE (2012). Comparative genomics of Shiga toxin encoding bacteriophages. BMC Genomics.

[CR37] Stolfa G, Koudelka GB (2012). Entry and killing of *Tetrahymena thermophila* by bacterially produced Shiga toxin. mBio.

[CR38] Wagner PL, Neely MN, Zhang X, Acheson DWK, Waldor MK, Friedman DI (2001). Role for a phage promoter in Shiga toxin 2 expression from a pathogenic *Escherichia coli* strain. J Bacteriol.

[CR39] Wagner PL, Livny J, Neely MN, David WK, Acheson DWK, Friedman DI, Waldor MK (2002). Bacteriophage control of Shiga toxin 1 production and release by *Escherichia coli*. Mol Microbiol.

[CR40] Waldor MK, Friedman DI (2005). Phage regulatory circuits and virulence gene expression. Curr Opin Microbiol.

[CR41] Węgrzyn G, Węgrzyn A (2005). Genetic switches during bacteriophage lambda development. Prog Nucleic Acid Res Mol Biol.

[CR42] Wegrzyn G, Glass RE, Thomas MS (1992). Involvement of the *Escherichia coli* RNA polymerase α subunit in transcriptional activation by the bacteriophage λ CI and CII proteins. Gene.

[CR43] Węgrzyn G, Licznerska K, Węgrzyn A (2012). Phage λ—new insights into regulatory circuits. Adv Virus Res.

[CR44] Werber D, Krause G, Frank C, Fruth A, Flieger A, Mielke M, Schaade L, Stark K (2012). Outbreaks of virulent diarrheagenic *Escherichia coli*—are we in control?. BMC Med.

